# Development of a GIS-based, real-time Internet mapping tool for rabies surveillance

**DOI:** 10.1186/1476-072X-5-47

**Published:** 2006-11-01

**Authors:** Jesse D Blanton, Arie Manangan, Jamie Manangan, Cathleen A Hanlon, Dennis Slate, Charles E Rupprecht

**Affiliations:** 1 Centers for Disease Control and Prevention, Atlanta, Georgia, USA; 2 Geospatial Research and Analysis Program, Agency for Toxic Substances and Disease Registry, Atlanta, Georgia, USA; 3 Warnell School of Forestry and Natural Resources, University of Georgia, Athens, Georgia, USA; 4 Wildlife Services, United States Department of Agriculture, Concord, New Hampshire, USA

## Abstract

**Background:**

Oral rabies vaccination programs have been implemented to control the spread of wildlife rabies in the United States. However, current surveillance systems are inadequate for the efficient management and evaluation of these large scale vaccine baiting programs. With this in mind, a GIS-based rabies surveillance database and Internet mapping application was created. This surveillance system, RabID, provides a new resource for the rapid mapping and dissemination of data on animal rabies cases in relation to unaffected, enzootic, and baited areas where current interventions are underway.

**Results:**

RabID is a centralized database for diagnostic and demographic information collected by local, state, and federal agencies involved in rabies surveillance. The geo-referenced database remits data to an Internet-accessible mapping application that displays rabies surveillance data in relation to environmental and geographic features.

**Conclusion:**

RabID provides a pioneering example of the power of geographically based Internet-accessible, infectious disease surveillance. This surveillance system was developed from existing technology and is readily adaptable to other infectious diseases and may be particularly useful for zoonoses. The development and application of public health informatics technology may enhance the effectiveness of public health interventions and allow better evaluation of public health interventions.

## Background

Rabies is an acute progressive viral encephalitis with a near 100% fatality rate. To date, no known treatment for rabies exists once symptoms of the disease become evident. Public education, vaccination of domestic pets, and the availability of human postexposure prophylaxis (PEP) have led to a decline of human rabies cases in the U.S. from 100's of cases a year prior to the 1950's to an average of 3 cases a year in the 21^st ^century [1-3]. However, the United States remains enzootic for wildlife rabies [4]. Human rabies cases are rare and the disease may not be clinically recognized, increasing the potential for novel transmission, as demonstrated by the organ transplantation cases in 2004 [5]. Although the number of human rabies cases is low each year, the number of persons potentially exposed and presenting for rabies PEP appears to have increased over the past decade with an estimate of approximately 40,000 persons per year in 1998 [6]. The currently estimated economic and public health cost for rabies in the United States is $300 million annually [7].

From a national perspective, rabies was one of the first diseases on the national notifiable disease list due to its public health importance. Over the past 60 years, the responsibility for national rabies surveillance has shifted to various U.S. agencies, from the Department of Agriculture (USDA) and its emphasis on dog rabies control and elimination, to the Department of Health and Human Services/Centers for Disease Control and Prevention (CDC) with its emphasis on human rabies prevention and control. However, the public health surveillance system is not intended nor sufficient to adequately characterize wildlife diseases that are zoonotic [8]. Comprehensive surveillance for wildlife zoonoses exceeds basic public health surveillance needs. Ideally surveillance for a zoonotic disease should consist of active sampling and precise location data, which are critical in identifying disease boundaries and intensities in relation to ecologic and land use features but are often independent of political boundaries. Animals are not randomly distributed and natural resources are not static, therefore, there is a strong spatiotemporal dynamic to zoonotic disease occurrence.

**Figure 1:**
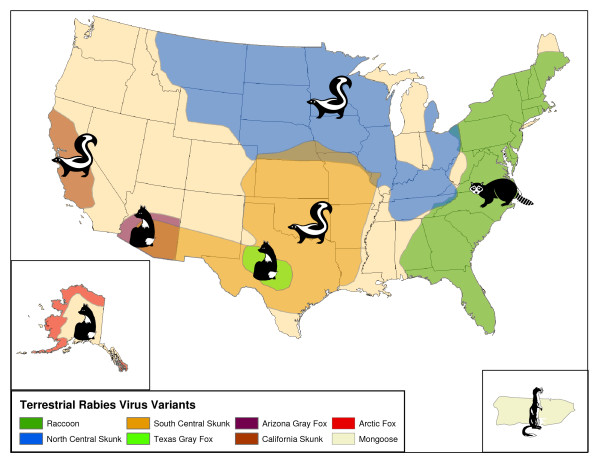
**Rabies Virus Reservoirs in the United States**. Geographic distribution of terrestrial rabies virus variants as defined by monoclonal antibody typing.

With the control and elimination of the canine rabies virus variant in the US, several geographically discrete terrestrial wildlife reservoirs were subsequently identified (figure 1). Today, raccoons are the most frequently reported rabid animal with more than 50,000 cases reported to the CDC from 1980 to 2000 [9]. The geographic spread of rabies among raccoons along the Eastern seaboard has had a major public health impact due to its overlap with high human population densities.

A novel method for the control of rabies in wildlife consists of the distribution of baits containing an oral rabies vaccine. The first field evaluation of a vaccine for raccoons occurred in the U.S. in 1990 [10]. Use of vaccine baits in an oral rabies vaccination (ORV) program appeared to have contributed to the elimination of a coyote rabies virus variant from Texas, perhaps in concert with drought conditions that limited coyote densities. Oral vaccination is currently being used as part of a more complex initiative to limit the spread of raccoon rabies and contain gray fox rabies in Texas [11].

With increasing emphasis being placed on ORV programs to control the spread of rabies, it has become apparent that current rabies surveillance programs are inadequate for the efficient management of these programs. In preparation for the anticipated 2002 start of a new ORV program in a region of Georgia, Alabama and Tennessee (later to be known as the GAT program), enhanced surveillance was initiated by the USDA/Wildlife Services (USDA/WS) to clarify and monitor the leading edge of the raccoon rabies front (particularly in Tennessee). Limitations in the existing surveillance reporting system led to discussions between state public health offices, USDA/WS, and CDC personnel in 2003. Consequently, a pilot project in the GAT region was instigated to develop an animal rabies surveillance system which would provide essential features for the management of the ORV programs, specifically:

- Integration of federal, state, and local surveillance activity and data

- Geographically precise location information

- "Real-time" access to surveillance data by participants and stakeholders

- The ability to display surveillance data geographically in relation to environmental features

- Adaptable to future surveillance needs

The development of this new rabies surveillance program was carried out by rabies laboratory personnel at CDC and the Geospatial Research, Analysis, and Services Program (GRASP) team of the Agency for Toxic Substances and Disease Registry (ATSDR). An information system for storing data was established and, in 2004, an Internet-accessible rabies mapping application was developed for online availability. The new system served as the central repository for data collected as part of ongoing enhanced surveillance by the USDA/WS as well as data routinely generated by state diagnostic laboratories as part of their rabies prevention activities. In 2005, four additional states began to participate in the project. As the surveillance system expanded from a regional to a national reporting system, it was named "RabID".

The RabID system provides rapid and timely analysis for the management and evaluation of the ORV programs in the United States. This paper outlines the architecture and capabilities of the RabID system, particularly its interactive mapping application. Also, future potential development of RabID and the obvious applicability of such a system to other infectious diseases will be discussed.

## Results

### Integration of multiple systems

Historically, national rabies surveillance consisted of aggregate data compiled from numerous, diverse local and state rabies diagnostic laboratories. For state and national case counts, data was obtained from various local and often disparate sources and reconciled into one system.

As of January 2006, RabID data originated from 16 laboratories in 7 states, representing routine (i.e. passive) public health surveillance from a total land area of 308,548 mi^2^. In addition, RabID incorporated data from enhanced surveillance conducted by USDA/WS personnel in 11 states with ORV programs. Enhanced surveillance consists of collecting suspected rabid animals which would not normally be tested due to a lack of human or domestic animal exposure (i.e. road kills, found dead, strange acting or nuisance animals).

### Components of RabID

#### -Database

Data is transmitted via email attachments in multiple and various formats (i.e. Microsoft Excel and Access). The data are then processed via an internally developed SAS macro (SAS Institute Inc., v9.0, Cary, NC, USA) which identifies and edits known formatting inconsistencies between the local data format and that of RabID.

After the data are processed in SAS, it is visually inspected by CDC personnel and records lacking a latitude-longitude coordinate are geocoded. Geocoding assigns geographic coordinates according to street address, zipcode, or county information contained within a spatial database. For RabID, geographic coordinates are interpolated according to a street-level-based geocoding system developed by the ATSDR, which references a high resolution database of roads that encompasses the entire United States.

Geocoded addresses are further checked in ArcGIS (ESRI Inc., v8.3, Redlands, CA, USA) to look for obvious errors in coordinate data (e.g. case is mapped outside the county of collection). The data are then imported into the RabID database, which resides on a SQL server.

#### -Spatial database

**Figure 2:**
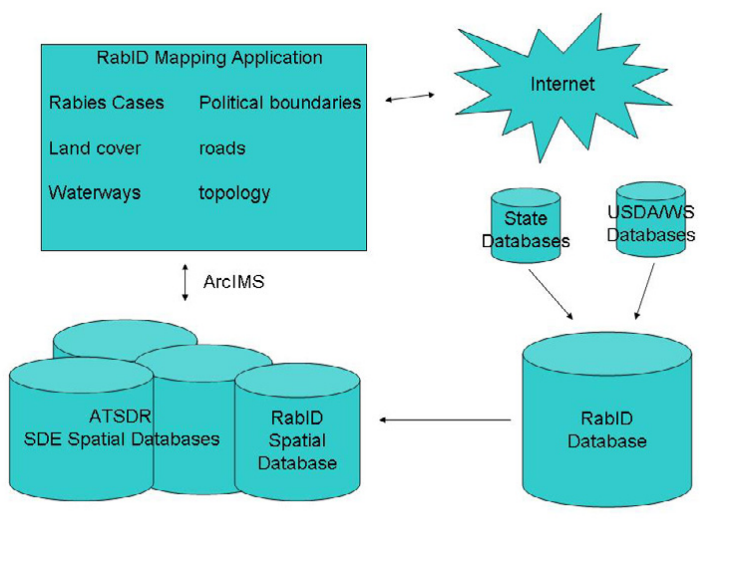
**RabID architecture**. General architecture of the RabID database and mapping application. (USDA/WS: US Department of Agriculture/Wildlife Services; ATSDR: Agency for Toxic Substances and Disease Registry).

Following addition of the data to the SQL server, the database is converted into an enterprise-level spatial database, which allows the RabID mapping application to maintain exclusive access for Internet-mapping functionality (figure 2). Essentially, the 'spatially enabled' database is identical to the original except that it contains spatial information beyond latitude and longitude (e.g. ESRI proprietary spatial information).

An alternative systematic approach would allow the mapping application to extract latitude and longitude coordinates directly from the RabID database for Internet-mapping purposes. However, due to the large size of the database (over 40,000 sample locations) and the constant addition of new cases, this approach is not feasible because a significant decrease in performance would result due to 'streaming' such a large dataset over the Internet. Our approach overcomes the performance issues inherent in the broadcast of a large database over a limited data stream by creating the spatial layer on the server rather than the client (i.e. web browser).

#### -Mapping application

**Figure 3:**
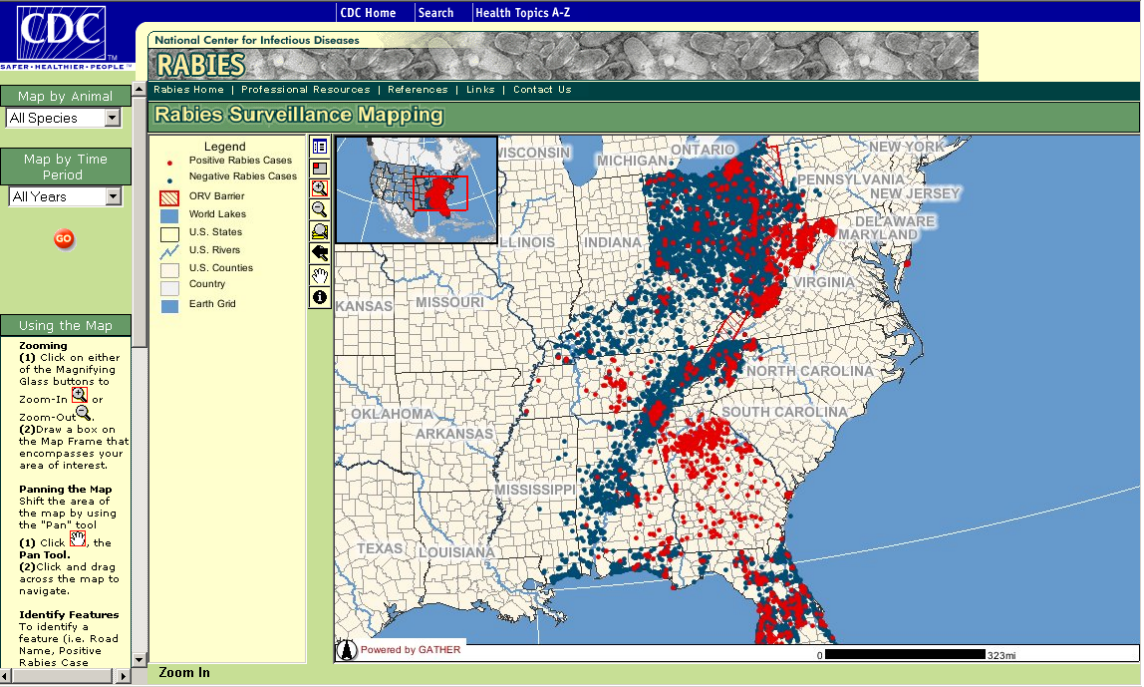
**RabID**. Complete data available in RabID to date. Blue indicates samples negative for rabies by laboratory diagnosis, red indicates positive.

**Figure 4:**
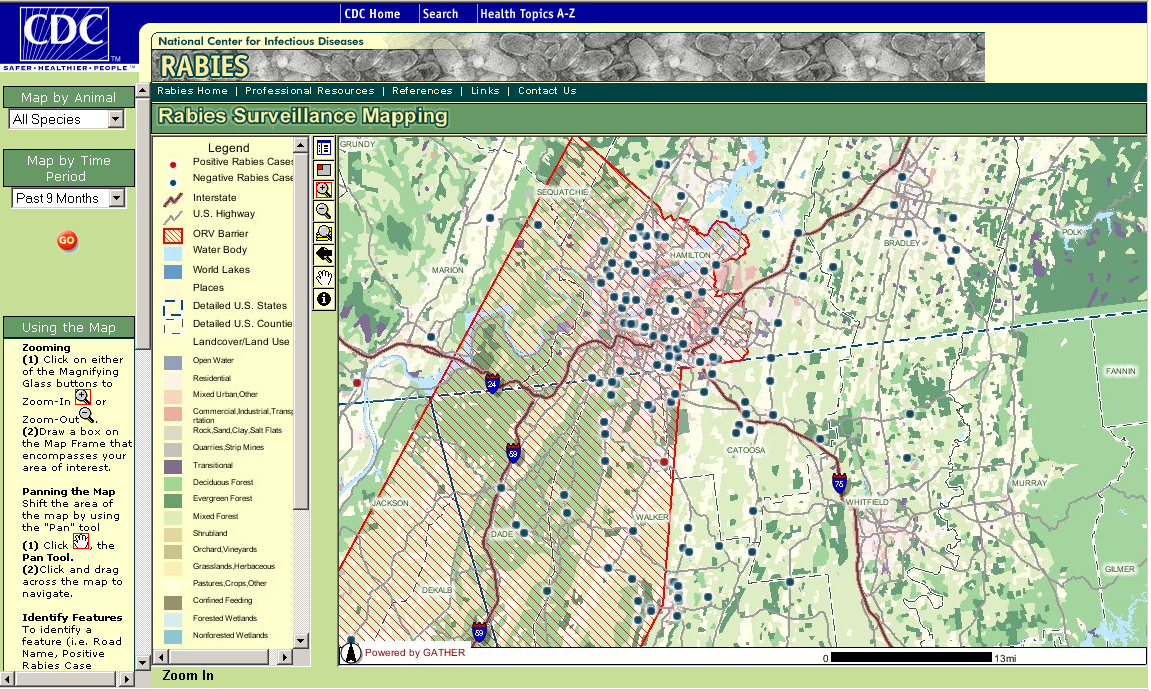
**RabID**. Zoomed view of rabies cases demonstrating additional environmental layers such as land use categories.

**Figure 5:**
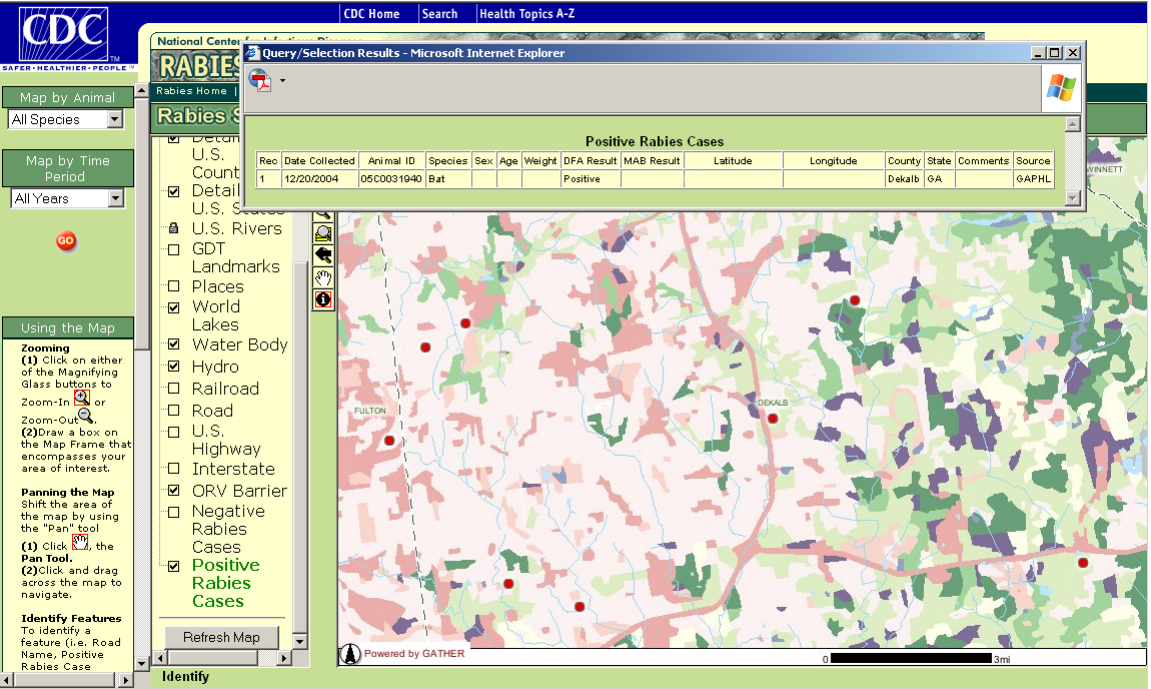
**RabID**. Zoomed view showing use of query tool to provide additional data on a rabies case.

The RabID mapping application is presented in a website to which access is password-protected and limited to cooperating federal, state, and local agencies. In addition to providing dynamic spatial interaction, RabID allows for temporal (e.g. yearly, quarterly, past three months) and animal specific (e.g. raccoon, skunk) querying of the database (figure 3). Animal locations are displayed according to their rabies diagnostic status in conjunction with other GIS data layers, such as land cover characteristics, rivers, lakes, streets and political boundaries (figure 4). Furthermore, individual cases can be queried via an 'identify' tool, which produces tabular data directly derived from the RabID spatial database (figure 5).

The RabID mapping application utilizes ArcIMS software (ESRI Inc., Redlands, CA, USA) and architecture to generate map images. When changes to the RabID maps are invoked on the client side by the user, a request is sent to the ArcIMS server which generates a new map image. The process model of the RabID ArcIMS application incorporates the ArcSDE database-schema (ESRI Inc., Redlands, CA, USA), which resides on a Microsoft SQL Server database (Microsoft Corp., Redmond, WA, USA). ArcSDE is an enterprise level relational database (i.e. allows for multiple users) that acts as a geographic data repository for GRASP. ArcIMS references the data layers (e.g. rabies cases, rivers, roads, land-cover) that reside on ATSDR's ArcSDE server to generate map images for the RabID mapping application.

### Future directions

RabID was initially developed to serve a small region along the Georgia, Alabama, and Tennessee borders. Its initial success and expansion to include additional states led to a reevaluation of the system and the need to increase its functionality. Redevelopment of the RabID system is currently ongoing to make the database architecture more susceptible to future variable additions, permit electronic web-based data entry, provide basic tabulation and reporting tools, and expand GIS functionality.

The current SQL database is being reformatted from flat file architecture to a relational database to allow for a more robust system as the amount of data increases and for easier addition of new variables. For example, a direct rapid immunohistochemical test (dRIT) has been developed as a new diagnostic tool for rabies detection under field conditions [12]. A new variable for dRIT results has recently been added to the database to reflect on-site preliminary rabies test results generated in the field by USDA/WS personnel.

The relational database architecture displays data by functional groups, for example, a demographic table (date of collection, animal species, age, sex, weight, collection method, samples collected, and if an exposure occurred), location table (coordinates, address, county and state), diagnostics table (DFA result, dRIT result, date of testing, and variant typing), serology table (RFFIT titer, and date of testing), and molecular table (sequence, primer set, date sequenced, and personnel conducting sequence). All data are linked by a unique animal identification number.

A web-based data entry method is currently under development to supplement the new database architecture and allow for the collection of greater amounts of data and additional participants. Although, in the early phase of development, it is envisioned that local data stewards will have direct access to their data for both primary entry and data correction or augmentation. In addition to capacity for single case entry a batch loading application will be developed to allow for the uploading of multiple records (e.g. daily or weekly compilations). Web-based tabulation tools are also under development to allow for basic reporting features to be performed on the website. Currently, table-based reporting has been developed for the tabulation of number and percent of cases reported by species and by state.

The Internet RabID mapping application is currently being rebuilt on a Microsoft .NET platform (Microsoft Corp., Redmond, WA, USA), to allow increased functionality and efficiency. Topographic data (e.g. United States Geologic Survey (USGS) 1:24,000 scale topographic maps) will be incorporated in the new version of the RabID application, along with the most updated land-cover classification available from USGS. Mapping functionality is also being expanded to include printing capabilities of the map display on the website.

## Conclusion

In comparison to other zoonotic diseases, rabies is currently unique in its potential for control and elimination in some terrestrial wildlife reservoirs through oral vaccination. The success of control programs depends heavily on timely and accurate disease surveillance. Many historically ingrained difficulties have been overcome by the development of this integrated and comprehensive rabies surveillance system. For example, in the past, rabies surveillance data consisted of positive case counts only, with no denominator data. With this practice, it was not possible to distinguish between an area with no rabies and an area with no animals submitted for testing (i.e. practicing a shoot-and-bury philosophy). Moreover, coarse location data (i.e. township or county) allows only a macro-analysis of rabies movement without capturing the fine details of correlation between animal host and environment.

One of the greatest achievements of this surveillance system is its "real-time" functionality. Public health rabies diagnostics is a time-efficient process because of its importance in guiding human rabies PEP. RabID acts as a mechanism to quickly disseminate the data to stakeholders. However, traditional enhanced surveillance carried out by USDA/WS, though vital for defining the leading edge of a rabies front, often has delays of up to four weeks due to the necessity of shipping samples to a laboratory, and the time required for testing large batches of samples. The development of the dRIT has allowed for enhanced surveillance activities to be performed at testing stations in the field decreasing the time-frame required for producing results. With the dRIT and RabID, it is now feasible for samples to be collected, processed, tested, and reported to all participants via the web in a single day. These advances provide an invaluable resource for decision making in regards to rabies control programs.

RabID represents one of the first multi-state, point location web-based surveillance systems (others include ArboNET and other West Nile Virus reporting systems). It is based on available technology which is readily adaptable to surveillance for other zoonotic diseases. Though confidentiality issues may be raised by the inclusion of human cases or exposure events, obfuscation and aggregation techniques are available that provide a method for presenting human cases of the disease overlaid by animal case point data [13]. This allows for direct correlation between animal and human disease burdens. The combined use of new laboratory techniques and informatics tools will allow for enhanced public health interventions as well as gaining insights into the epidemiology of rabies and other diseases.

## Competing interests

The author(s) declare that they have no competing interests.

Use of trade names and commercial sources are for identification only and do not imply endorsement by the U.S. Department of Health and Human Services.

## Authors' contributions

JB currently maintains the RabID system and prepared the draft of this manuscript. AM drafted the technical nature of the manuscript and proof read for accuracy. JM, CH, DS and CR assisted in the preparation of the manuscript and are part of the development team for the RabID system.
